# Nociceptive pain in adult patients with 5q-spinal muscular atrophy type 3: a cross-sectional clinical study

**DOI:** 10.1007/s00415-022-11351-0

**Published:** 2022-08-29

**Authors:** Elena Sagerer, Corinna Wirner, Benedikt Schoser, Stephan Wenninger

**Affiliations:** Department of Neurology, Friedrich-Baur-Institute, Ludwig-Maximilians University Munich, Ziemssenstr. 1, 80336 Munich, Germany

**Keywords:** Nociceptive pain, Spinal muscular atrophy, SMA3, Clinical outcome, Pain pressure threshold, Myotonometry

## Abstract

**Background:**

Spinal muscular atrophy (SMA) is an autosomal recessive neuromuscular disorder caused by mutations in the SMN gene, leading to progressive muscular weakness, atrophy and so far neglected musculoskeletal pain. This study is the first to characterize nociceptive pain in patients living with SMA type 3 by assessing whether muscle pain is associated with alterations in muscle strength, function, stiffness, frequency, decrement, relaxation, or creep.

**Methods:**

We performed a cross-sectional pilot study on 20 SMA3 patients. We evaluated motor function and muscle strength (dynamometry, quick motor function test and 6-min-walk test), nociceptive pain (pressure algometer evaluating muscular pressure pain threshold (PPT)) and non-invasive measurement of muscle stiffness, frequency, decrement, relaxation, or creep (myotonometry with the MyotonPro^®^). For statistical analysis, we used *t* tests, Mann–Whitney *U* tests and linear regression.

**Results:**

Significantly more women than men reported musculoskeletal pain (*p* = 0.003). A lower score in dynamometry was associated with lower scores in PPT in all extremities reflecting a higher sensitivity of these muscles to pressure. We did not find significant correlations between the PPT values and the MyotonPro values in the corresponding muscles. Assessments of PPT before and after the 6-min walk test did not show clinical meaningful changes. Besides nociceptive pain, fatigue was prevalent in 50% and pain in 55% of the patients.

**Conclusions:**

Muscle pain in SMA3 is associated with muscular weakness in the arms and legs, but not with changes in muscular stiffness, frequency, decrement, relaxation, or creep. This shows that muscle pain in SMA3 is mainly caused by changes in the dysbalanced musculoskeletal system due to muscle weakness.

**Supplementary Information:**

The online version contains supplementary material available at 10.1007/s00415-022-11351-0.

## Introduction

Spinal muscular atrophy (SMA) is a rare autosomal recessive neuromuscular disorder caused predominantly by a homozygous deletion [24] of the SMN1 gene on chromosome 5q13 [11] with the absence of SMN1 in exon 7 [4]. The number of compensatory increased SMN2 copies influences the clinical phenotypes, formerly classified as types 0–4 [17]. Since 2017, three specific therapeutic agents have been approved, including nusinersen, onasemnogene abeparvovec (only for SMA type 1), and risdiplam [7, 13]. As a result of novel specific therapies, the new classification was necessary to reflect the clinical phenotypes more appropriately and now subdivided into “non-sitter”, “sitter” and “walker” [12]. In SMA type 3 or walkers, patients develop a slowly progressive proximal muscle weakness and muscular atrophy to a variable degree and rarely restrictive respiratory insufficiency.

The prevalence of pain and fatigue is estimated between 30 and 90% in all types of neuromuscular diseases [6]. Musculoskeletal pain may appear to variable degrees as a primary symptom, but also evolve frequently with the progression of the disease. It is unclear whether the pain is caused by the disease itself or whether ancillary factors such as muscular imbalance or increased muscle tension contribute to amplified pain perception. Nevertheless, pain has a considerable impact on quality of life and disease burden and is often treated negligibly. In SMA type 3 or walkers, with the progression of muscular weakness, nociceptive pain occurs in some patients, but clinical studies on pain in these SMA patients are rare. Few recommendations have been published, mainly focusing on pain caused by skeletal deformities and orthopedic corrections [18]. Besides, some surveys have included questions regarding pain in different SMA phenotypes [2, 5, 10]. This is the first clinical study evaluating nociceptive pain in SMA type 3 (walkers).

## Methods

### Study setting and patient population

This explorative, cross-sectional clinical study aimed to characterize the prevalence, quality, intensity and distribution of musculoskeletal nociceptive pain, and fatigue in adult patients with longstanding SMA3. The second objective was to assess whether muscle pain severity and distribution are associated with alterations in muscle function, muscle strength and muscle stiffness or elasticity. Only adult patients aged ≥ 18 years with genetically confirmed 5q-SMA, clinically type 3/walkers and ability to perform study-related functional tests were enrolled in this study. Because of possible interference between depressive symptoms and pain, patients with a Beck Depression Inventory-Fast Screen score > 3 were excluded from study participation. Other exclusion criteria were participation in another clinical study, use of an investigational treatment, or inability (in the investigator's opinion) to adhere to the requirements of the study. All patients were enrolled at the neuromuscular expert center Friedrich-Baur-Institute at the LMU Munich, Germany. The study was approved by the ethics committee of the LMU Klinikum, Project No. 20–0980, and the protocol was registered on a public clinical trials registry (ClinicalTrials.gov Identifier NCT04907162).

### Examinations and methods

Musculoskeletal strength was assessed by the MRC scale (Medical Research Council scale) and dynamometry. Muscle strength was assigned an MRC score with a range from 0 (no movement possible) to 5 (maximum force) for each muscle [3]. Dynamometry was used to document maximum force in kg as the best of three attempts to ensure a high level of objective and precise results. The neck flexor and extensor, shoulder abductor, elbow flexor and extensor, hand extensor, finger flexor, hip flexor, knee flexor and extensor, and foot flexor and extensor, each on the left and right sides were examined.

A pressure algometer was used as a reliable method to quantify local pain by measuring the musculoskeletal pressure pain threshold (PPT) in different muscles. The algometer was placed onto the muscles with a contact area of 1 cm^2^ and the stimulus intensity was increased until the patient felt any sensation of pain. The result was taken in kg as the arithmetic mean of three repeats and was recorded on both sides of the trapezius, deltoideus, supraspinatus, biceps brachii, rectus femoris, tibialis anterior and gastrocnemius muscles, as well as the erectors spinii and the neck extensor muscles.

A 6-min walk test (6MWT) and a Quick Motor Function Test (QMFT) were performed to examine the physical function and muscular endurance. The 6MWT [1] was adapted for this study by adding algometer measurements at the lower limbs (*M. rectus femoris* and *M. gastrocnemius*) before and after the 6MWT and evaluating pain symptoms during the test. The QMFT is widely used in assessing neuromuscular diseases, delivering a score of movement and muscle function from 0 to 64 points [22].

For the non-invasive measurement of muscle stiffness, muscle tone, muscle relaxation and viscoelasticity, myotonometry was performed using the commercially available MyotonPro^®^. The tip of the MyotonPro^®^ is placed perpendicular to the underlying muscle. A slight pressure with a constant preload induces an oscillation, which leads to a short-term deformation of the underlying muscle. An acceleration sensor now analyzes the vibration behavior of the muscle and the viscoelastic stiffness is calculated. The calculation is based on the stiffness of the muscle (in N/m), the elasticity (the ability of the muscle to return to its initial shape) and the duration required for this. Values of the MyotonPro were assessed for the neck extensor muscles, the trapezius, supraspinatus, deltoideus, biceps brachii, erectors spinii, rectus femoris, tibialis anterior and gastrocnemius muscles on both sides. The MyotonPro^®^ was rated as having an excellent test–retest reliability for most muscles [3], but was hardly described in any neuromuscular disease.

### Disease-related history and pain profile

The characteristics of both pain types were explained to the patient to differ between nociceptive and neuropathic pain. Pain that could not be assigned to nociceptive pain (i.e., mixed pain, often experienced in the back) was also captured in the study. Before the clinical examination, disease-related history, medical history and disease- and quality-of-life-related questionnaires (FSS, BPI and German Pain Questionnaire) were obtained. Historical data were collected with a focus on the intensity, frequency, and quality of nociceptive pain and previous and current drug use or non-drug therapy methods (physiotherapy, manual therapy) and surgery influencing the myalgia. The Fatigue Severity Scale (FSS) includes nine questions and evaluates the severity of fatigue, i.e., physical and mental exhaustion and its influence on the quality of life. The FSS total score is the average of the nine-item scores and ranges from 1 (“no signs of fatigue”) to 7 (“most disabling fatigue”). For this study, a cutoff value greater than four was chosen for evaluating the presence of fatigue [20]. The FSS was originally designed for people with multiple sclerosis or systemic lupus erythematosus [9] and is also currently used for the evaluation of fatigue in various neuromuscular diseases such as Pompe disease [8]**,** spinal muscular atrophy type 2 and congenital myopathies [23]. The Brief Pain Inventory is a questionnaire on the severity of pain and impairment of function caused by the pain in the past 24 h. The questionnaire consists of a map of the human body on which the patient should mark the distribution of the nociceptive pain. Furthermore, the results of the BPI are two scores [16]. The pain intensity score is calculated using four items on pain intensity (strongest pain, lowest pain, average pain, pain at the moment). Each item is rated by the patient from 0 (no pain) to 10 (worst imaginable pain) and contributes with the same weighting to the final score (0–40 points). For a pain interference score, seven sub-points (general activity, mood, ability to walk, normal gait, relationships with other people, sleep and quality of life) are rated from 0 (not disruptive) to 10 (completely disruptive) and contribute with the same weighting to the final score (0–70 points). The BPI is a widely used questionnaire on pain [16] and is internationally recommended for the assessment of chronic pain in various clinical trials [21].

A detailed description of pain was evaluated with the German Pain Questionnaire (GPQ), including pain sites, duration, intensity, pain-relieving and -aggravating conditions and subjective pain perception [14]. The GPQ is based on a bio-psycho-social pain model and therefore includes questions regarding the prevalence of depression, fear and stress, comorbidities, social situation and disease-related quality of life. The GPQ has been developed and validated by the Taskforce on “Standardization and Economy in Pain Management” of the German Chapter of the International Association for the Study of Pain (DGSS) [19].

The DASS (Depression, Anxiety and Stress Scale) is a brief and reliable questionnaire [15]. The questionnaire contains 21 questions, 7 items each for depression, anxiety and individual stress. In comparison to the BDI, which was designed for a psychiatric assessment of depression, the questions are worded less drastically, so patients are less deterred by them. Therefore, sensitivity to weaker psychiatric disorders is higher on the DASS. For each of the three scales, cutoff values were set for an increased likelihood of the presence of depressive disorder (cutoff: > 10 points), anxiety disorder (cutoff: > 6 points) or increased stress load (cutoff: > 10 points).

The Marburg Questionnaire (MFHW) was used to assess habitual well-being in relation to perceived pain. It consists of seven questions regarding mental, physical and emotional well-being and performance despite the experienced pain. It provides additional information compared with the DASS by addressing positive abilities of the patients. The maximum score is 35 points, indicating a particularly high level of well-being. A score of ten points and below is a low value of habitual well-being in pain patients.

A detailed description of pain was evaluated with the German Pain Questionnaire (GPQ), including pain sites, duration, intensity, pain-relieving and -aggravating conditions and subjective pain perception [14]. The GPQ also included questions regarding the prevalence of depression, fear and stress, comorbidities, social situation and disease-related quality of life.

### Statistical analysis

SPSS Statistics^®^ Version 27 and Microsoft Excel^®^ 2016 were used for the analysis. For the illustrations of the human body, BioRender^®^ was used. All data were graphically checked for normal distribution. For all metric, normally distributed values, we performed an unpaired, two-sided *t* test. For all non-parametric values, we performed a Mann–Whitney *U* test. For the change in PPT values before and after the 6MWT, we performed a paired two-sided *t* test or a Wilcoxon rank test. The significance level (alpha) was set at ≤ 0.05.

## Results

All patients gave written informed consent before the first assessment. Of 25 patients screened for eligibility, 20 patients were enrolled in the study. Two patients declined to participate after study information, and three patients were not eligible due to a score of ≥ 4 in BDI-Fs at screening. Because of the significantly different perceptions of pain in men and women (Table 1), we divided the SMA patients into a male and a female subgroup to identify different sex-dependent pain patterns.

**Table 1:** Demographic characteristics of the enrolled 5Q-SMA3 patients

	Male	Female	Total	*p* value
Number (percent)	*N* = 13(65%)	*N* = 7(35%)	*N* = 20	0.18
Age at baseline (years)				
Mean (± SD; min; max)	40.15 (± 12.48; 20; 57)	34.71(± 14.74; 20; 54)	38.25 (± 13.19; 20; 57)	0.39
Age at first symptom (years)				
Mean (± SD; min; max)	12.54 (± 9.85; 2; 40)	7.29 (± 4.68; 2; 14)	10.70 (± 8.65; 2; 40)	0.20
Age at diagnosis (years)				
Mean (± SD; min; max)	18.15 (± 13.2; 4; 51)	18.57 (± 15.43; 5; 48)	18.30 (± 13.64; 4; 51)	0.95
Disease duration (years)				
Mean (± SD; min; max)	27.62 (± 13.05; 9; 53)	27.43 (± 13.35; 11; 49)	27.55 (± 12.80; 9; 53)	0.938
SMN2 copies				
Mean (± SD; min; max)	3.77 (± 0.44; 3; 4)	3.71 (± 0.49; 3; 4)	3.75 (± 0.44; 3; 4)	0.80
BMI*				
Mean (± SD; min; max)	24.24 (± 5.01; 17.5; 35.5)	25.26 (± 4.27; 20.5; 31.2)	24.60 (± 4.68; 17.5; 35.5)	0.66
Mobility				0.154
Ambulant	*N* = 7 (54%)	*N* = 6 (86%)	*N* = 13 (65%)	
Non ambulant	*N* = 6 (46%)	*N* = 1 (14%)	*N* = 7 (35%)	
Disease-specific therapy				
Nusinersen	*N* = 13 (100%)	*N* = 7 (100%)	*N* = 20 (100%)	
Therapy since (years)	3.07 (± 0.65; 2; 4,1)	3.05 (± 0.57; 2.5; 4.1)	3.06 (± 0.61; 2; 4.1)	0.877
Start at age	37.0 (± 12.4; 18; 54)	31.3 (± 15.5; 16; 52)	35 (± 13.5; 16; 54)	0.241
Report of pain (historical)	*N* = 4 (31%)	*N* = 7 (100%)	*N* = 11 (55%)	0.003*
Nociceptive	*N* = 2	*N* = 4	*N* = 6	
Mixed	*N* = 1	*N* = 4	*N* = 5	
Chronic pain (> 6 months)	*N* = 4 (31%)	*N* = 6 (86%)	*N* = 10 (50%)	
Pain during 6MWT	*N* = 0 (0%)	*N* = 2 (28.6%)	*N* = 2 (10%)	0.202
Pain-specific therapy**	*N* = 0 (0%)	*N* = 2 (28.6%)	*N* = 2 (28.6%)	0.060
Number of body parts affected by pain	1.5 ± 0.6	3.9 ± 2.6	3 ± 2.4	
FSS points***	3.955	4.114	4.011	0.699
Presence of fatigue***	*N* = 6 (46%)	*N* = 4 (57%)	*N* = 10 (50%)	0.64
QMFT				
Mean (± SD; min; max)	27.23 (± 15.09; 5; 56)	34.83 (± 14.13; 10; 53)	29.63 (± 14.85; 5; 56)	0.313
MRC sum score****				
Mean (± SD; min; max)	82.5 (± 16.3; 51; 108.5)	91.4 (± 11.24; 77; 105.5)	85.63 (± 15.07; 51; 108.5)	0.215

^*^BMI = weight[kg]/(height [m])^2^; **All patients took ibuprofen 600 mg one to four times a month as pain-specific treatment. ***Points in the Fatigue Severity Scale (1–7 points); for the presence of fatigue, a cutoff > 4 points was chosen; ****MRC sum score was calculated by summing MRC of 12 muscles or regions (both sides), ranging from 0 (complete paralysis) to 110 (normal strength): neck flexors and extensors, deltoid, biceps brachii, triceps brachii, hand extensors, finger flexors, iliopsoas, knee flexors and extensors as well as foot flexors and extensors, each of them ranged from 0 (complete paralysis) to 5 (normal strength)

All significant values are bold

### Baseline demographics and characteristics

Baseline demographics are provided in Table 1. All 20 patients (13 male, 65%) had genetically confirmed 5q spinal muscular atrophy. At baseline, the median age was 40.5 years, age at first symptom 11 years and age at diagnosis 16 years. No statistically significant differences were found between men and women regarding baseline demographics, reflecting an equal distribution. Four men (31% of the male cohort) and all seven women reported pain (*p* = 0.003). Two patients (26.8%) took pain medication irregularly as needed due to muscle pain. The pain was chronic (experienced for longer than 6 months) in ten patients. The pain was only nociceptive in six patients and mixed in five patients. All patients received nusinersen as a specific disease treatment. 13 (65%) patients were ambulant and could perform the 6MWT. In seven (35%) patients who were either wheelchair dependent or only able to walk a few steps, the 6MWT was not performed. There was no significant difference between men and women in any category regarding muscle strength, muscle function (assessed by the QMFT) or fatigue.

### Fatigue

Prevalence of fatigue (FSS score > 4) was high within the cohort (50% of all patients) and was present in six male (46%) and four female (57%) patients.

### Pain frequency and distribution

**Fig. 1:**
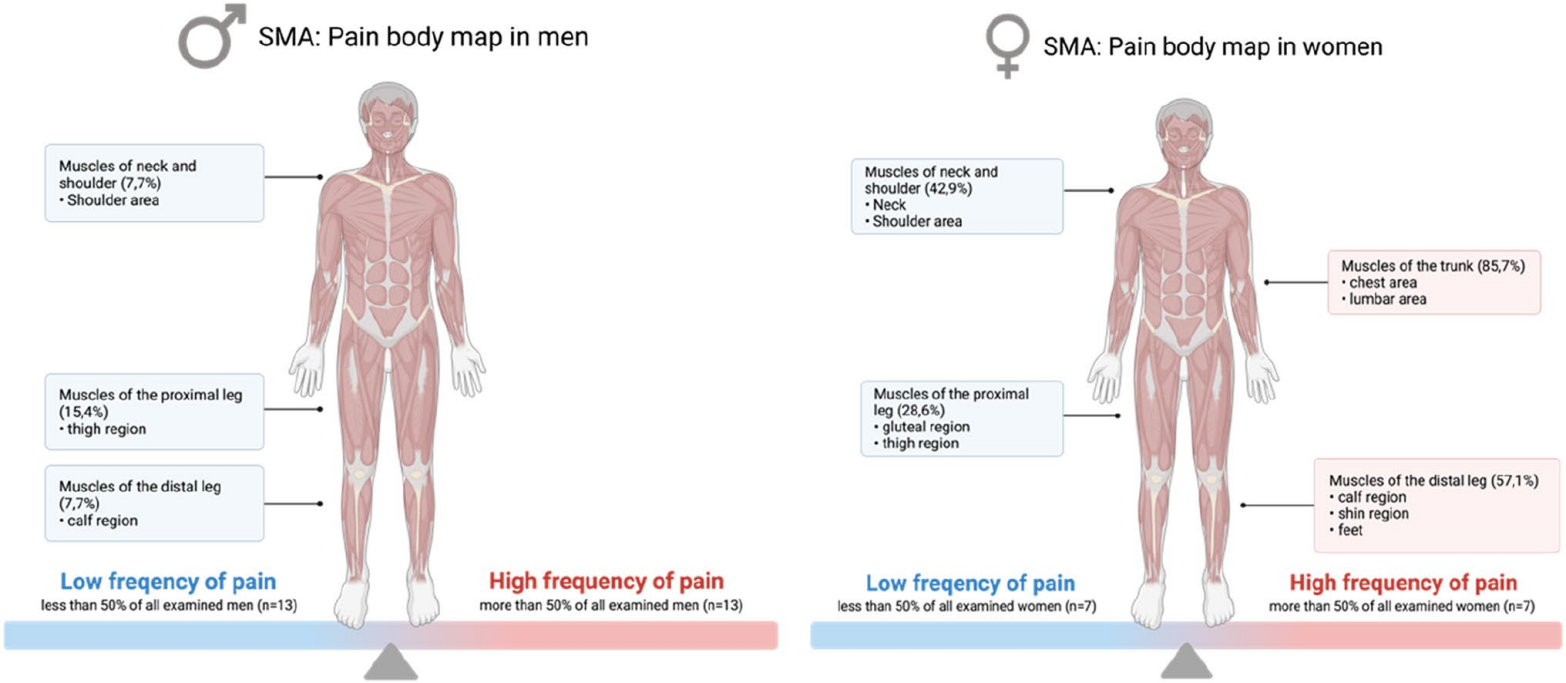
Frequency of pain in all examined men (*N* = 13) and women (*N* = 7)

**Fig. 2:**
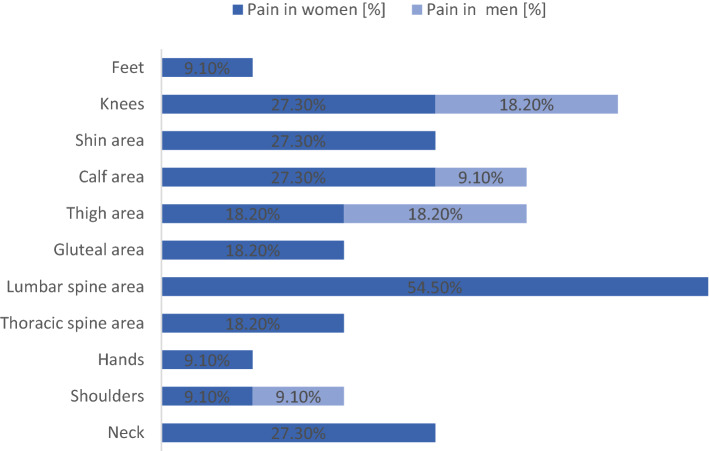
Location of perceived pain in all patients reporting pain (*n* = 11). Percentage of patients experiencing pain in different locations of all 11 patients reporting pain

55% (*n* = 11) of the SMA patients reported nociceptive or mixed pain with a high localization and strength variability, visualized in Figs. 1 and 2. Most of the patients reporting pain suffered from pain attacks (*n* = 9, 81.8%), and only two patients reported permanent pain (18.2%). Overall, the pain was perceived in declining frequency in the lumbar spine region (54.5%), the legs, especially in the knees (45.5%) and in the neck (27.3%)**.**

Women with SMA experienced pain significantly more often (*p* = 0.003; Table 1), and the distribution of pain differed between men and women (Figs. 1 and 2). In women, the pain was reported most frequently in the trunk/chest muscles, lumbar region, distal leg muscles, and less frequently in the shoulder and neck muscles and proximal leg muscles. Men reported pain less regularly and only in the shoulders and legs (proximal and distal). Furthermore, women experienced pain in a higher number of various regions of their body (3.9 ± 2.6 different locations in women versus 1.5 ± 0.6 other locations in men; Table 1).

### Pain quality and intensity

Pain characteristics are reported in Table 2. Most frequently, the pain was described as pulling, oppressive and dull. In 81.8%, the pain was described as pain attacks with a duration of minutes (45.5%) or seconds (18.2%). In 54.5%, no daily dependency was reported. Pain severity was rated as mild to moderate on a scale from 0 to 10: Within the last 4 weeks, the maximum pain severity evaluated by the numeric rating scale (NRS) was 3.91 (SD ± 2.12), and the mean pain score was 1.73 (SD ± 1.19). The pain severity score in the last 48 h, evaluated by BPI, was higher in women (4 ± 3.8) than in men (2.8 ± 1.3), whereas the pain interference score was higher in men (11.5 ± 13.9) than in women (7.1 ± 9.8). There were also differences in the categories of interference that were most affected by the pain (on an interference scale from 0 to 10; 0 = no interference, 10 = high interference). In women, enjoyment of life (1.43), activity (1.43) and general mood (1.29) were most affected by pain. In men, interferences with walking (3), work (2.25), enjoyment of life (2), relationships (1.5) and sleep (1.5) were most affected.

**Table 2:** Pain characteristics

Pain characteristics	Pain in women (*n* = 7)	Pain in men (*n* = 4)	Pain in men and women (*n* = 11)
Pain severity score mean (last 48 h) (0–40)	4 ± 3.8	2.8 ± 1.3	3.5 ± 3.1
Pain interference score mean (last 48 h) (0–70)	7.1 ± 9.8	11.5 ± 13.9	87 ± 11
Pain severity score mean (last 4 weeks)			
Highest score	4.29 ± 1.89	3.25 ± 2.63	3.91 ± 2.12
Mean score	2.00 ± 0.58	1.25 ± 1.89	1.73 ± 1.19
Pain characteristics			
Emotional characteristics	57%	25%	46%
Dull	71%	50%	64%
Oppressive	86%	50%	73%
Throbbing	43%	25%	36%
Knocking	29%	25%	27%
Stabbing	57%	25%	36%
Pulling	86%	100%	91%
Hot	14%	0%	9%
Burning	29%	50%	36%
Impairment (score from 1 to 10) of			
Ability to walk	1.00 ± 1.92	0.92 ± 2.25	1.73 ± 2.61
Normal gait	1.00 ± 1.67	0.69 ± 1.70	1.45 ± 1.86
Sleep	1.00 ± 1.53	0.46 ± 1.66	1.18 ± 2.04
Enjoyment of life	1.43 ± 2.94	0.62 ± 1.50	1.64 ± 2.62
Relationships with other people	0.00 ± 0.00	0.46 ± 1.39	0.55 ± 1.51
General activity	1.43 ± 2.15	0.15 ± 0.38	1.09 ± 1.76
Mood	1.29 ± 2.98	0.23 ± 0.60	1.09 ± 2.39
Fluctuations and attack durations			
Permanent pain	28.6% (*N* = 2)	0%	18.2% (*N* = 2)
Pain attacks	71.4% (*N* = 5)	100% (*N* = 4)	81.8% (*N* = 9)
Attack duration			
Seconds	28.6% (*N* = 2)	0%	18.2% (*N* = 2)
Minutes	14.3% (*N* = 1)	100% (*N* = 4)	45.5% (*N* = 5)
Hours	14.3% (*N* = 1)	0%	9.1% (*N* = 1)
Up to 3 days	14.3% (*N* = 1)	0%	9.1% (*N* = 1)
Attack frequency			
Several times daily	14.3% (*N* = 1)	25% (*N* = 1)	18.2% (*N* = 2)
Daily	14.3% (*N* = 1)	0%	9.1% (*N* = 1)
Weekly	14.3% (*N* = 1)	75% (*N* = 3)	36.4% (*N* = 4)
Monthly	14.3% (*N* = 1)	0%	9.1% (*N* = 1)
Less often	14.3% (*N* = 1)	0%	9.1% (*N* = 1)
Daily dependance			
None	28.6% (*N* = 2)	100% (*N* = 4)	54.5%
In the morning	28.6% (*N* = 2)	0%	18.2%
In the evening	14.3% (*N* = 1)	0%	9.1%
In the night	28.6% (*N* = 2)	0%	18.2%
Time since onset of pain			
< 1 month	14.3% (*N* = 1)	0%	9.1% (*N* = 1)
1 month to ½ year	0%	0%	0%
1/2 year to 1 year	0%	25% (*N* = 1)	9.1% (*N* = 1)
1 year to 2 years	14.3% (*N* = 1)	0%	9.1% (*N* = 1)
2 year to 5 years	28.6% (*N* = 2)	0%	18.2% (*N* = 2)
> 5 years	42.9% (*N* = 3)	75% (*N* = 3)	54.5% (*N* = 6)

### Physical and mental comorbidities

Comorbidities and habitual well-being are presented in Table 3. Although women experienced pain significantly more often and with a higher severity, women experienced higher habitual well-being than men (27.4 ± 6.3 vs 21 ± 11). Results of the DASS showed lower values for depression and stress disorders and equal values for anxiety disorders in women with pain compared to men with pain. However, men without pain reported the lowest values for depression, stress and anxiety. Comorbidities were more common in men than in women, especially cardiovascular and gastrointestinal disorders. One male and two female patients with nociceptive pain had scoliosis, and two women reported cervical spine stenosis. 100% of the women regularly attended physical therapy, compared to 75% of the men with pain and 56% of the men without pain.

**Table 3:** Physical and mental comorbidities, physiotherapy and general well-being in women and men with and without pain

	Women with pain (*n* = 7)	Men with pain (*n* = 4)	Men without pain (*n* = 9)
MFHW* (mean ± SD, min; max)	27.4 ± 6.3; 18; 34	21 ± 11; 10; 32	–
Depression score (mean ± SD, min; max)	2.1 ± 1.8; 0; 5	3.3 ± 3.2; 1; 7	1.1 ± 2.4; 0; 7
Anxiety score (mean ± SD, min; max)	2.7 ± 3.9; 0; 11	2.7 ± 1.5; 1; 4	1.9 ± 2.4; 0; 7
Stress score (mean ± SD, min; max)	4.1 ± 4.1; 0; 11	5.3 ± 5.8; 2; 12	2.5 $\pm$ 4.4; 0; 13
Physiotherapy	100%	75%	56%
Comorbidities			
Musculosceletal	57% (*n* = 4)	25% (*n* = 1)	–
Cardiovascular	–	–	22.2% (*n* = 2)
Gastrointestinal	–	25% (*n* = 1)	11.1% (*n* = 1)
Skin	–	25% (*n* = 1)	–

Musculoskeletal comorbidities in women with pain: cervical spine stenosis (*n* = 2), scoliosis (*n* = 2)

Musculoskeletal comorbidities in men with pain: scoliosis (*n* = 1); Other comorbidities in men: cardiovascular: hypertension (*n* = 2); gastrointestinal: diverticulosis (*n* = 1) and reflux disease (*n* = 1); skin: psoriasis (*n* = 1)

*MFHW (Marburg questionnaire on habitual well-being despite the perceived pain): maximum (35 points) means particularly great well-being

### Pain triggers and relievers

Pain triggers are summarized in Table 4. Most frequently, the pain was triggered by doing too much exercise or physical exertion, such as running or walking for too long (63.6%; *N* = 7). To relieve the pain, most patients chose relaxation, for example, going for a walk or resting (72.7%; *N* = 8). In general, movement and exercises were associated with pain triggers and pain relievers. Two female patients used pain-specific treatment: both took ibuprofen 600 mg as needed up to four times a month. One female patient tried massage and hot baths for pain relief, but with only minimal benefit.

**Table 4:** Pain-relieving and -aggravating conditions

	Women and men experiencing pain (*N* = 11)
Pain-aggravating conditions	
Too much exercise and physical exertion, i.e., running or walking too much	63.6% (*N* = 7)
Wrong posture	27.3% (*N* = 3)
Falls	18.2% (*N* = 2)
Wrong moves	18.2% (*N* = 2)
Immobility	9.1% (*N* = 1)
Overweight	9.1% (*N* = 1)
Pain-relieving conditions	
Relaxation, i.e., going for a walk or resting	72.7% (*N* = 8)
Strengthening the muscles by doing specific exercises such as physiotherapy	36.4% (*N* = 4)
Warmth	18.2% (*N* = 2)
Distraction, i.e., reading a book	18.2% (*N* = 2)

### SMA-specific treatment

All patients received nusinersen as disease-specific treatment at baseline (starting at age 35 with a mean therapy duration of 3.06 years; Table 1). None of the patients reported an impact on pain or fatigue occurrence, neither a change in frequency, intensity, characteristics or fluctuation of pain.

### Objective assessments: PPT, dynamometry and MyotonPro^®^ results

#### Pain pressure threshold values and muscle strength

**Fig. 3:**
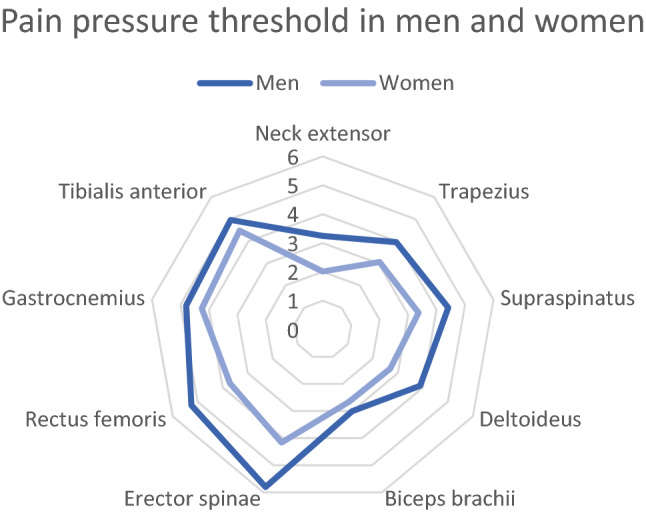
PPT values [kg] in men (*N* = 13) and women (*N* = 7). Mean force, displayed in kilogram, needed to induce pain in the muscle. The patients were separated into a female (*n* = 7) and a male (*n* = 13) subgroup. Nine different muscles were examined on both sides

Pain pressure threshold (PPT) was lower in women than in men in nearly all muscles (Fig. 3). However, the differences between men and women were only slightly statistically significant in the M. rectus femoris on the left side (*p* = 0.037; Supplements Table 1), which was no longer evident after Bonferroni correction (significance level was set at alpha = 0.003). Figure 3 highlights the force needed to induce pain in men compared to women, displayed for nine different muscles.

For a linear regression model, we summarized PPT values of four body regions: the arms (deltoid and biceps brachii), legs (rectus femoris, tibialis anterior and gastrocnemius muscles), shoulder and neck region (supraspinatus, trapezius and neck extensor muscles) and trunk (erector spinii muscles) (Table 5). We also summarized the dynamometer scores for an arm (dynamometer values for the biceps brachii, the triceps brachii and the deltoid muscles) and a leg region (hip flexion, knee extension and knee flexion, foot extension and foot flexion). Because the legs were particularly affected in the patients, we divided the leg region into a proximal and a distal part. Data were visually scanned for autocorrelation and homoscedasticity. Pressure pain threshold values show a positive regression for the arm (*p* = 0.041*) and the leg region (*p* = 0.024*), especially for the distal part (*p* = 0.022*), but not for the shoulder and neck region or for the trunk region (Table 2). A lower score in dynamometry was associated with lower scores in PPT in the extremities, reflecting a higher sensitivity of these muscles to pressure.

**Table 5:** Linear regression for PPT (pressure pain threshold) as a dependent variable

PPT summarized [kg]	Dynamometer arm [kg]	MRC trunk	MRC neck extensor	Dynamometer leg [kg]	*p* (model)	*R*	*R*^2^	Adjusted *R*^2^	Regression model
Arm region	0.041*	–	–	–	0.041*	0.461	0.213	0.169	11.119 + 0.065 * x
Trunk region	–	0.862	–	–	–	–	–	–	–
Leg region	–	–	–	0.024*	0.024*	0.561	0.315	0.266	21.158 + 0.157 * x
Proximal leg region	–	–	–	Proximal leg values: 0.159	–	–	–	–	–
Distal leg region	–	–	–	Distal leg values: 0.022*	0.022*	0.568	0.323	0.275	13.258 + 0.139 * x
Shoulder and neck region	0.127	–	0.650	–	–	–	–	–	–

A linear regression model was performed to identify a relationship between pressure pain threshold values and muscle strength in different body regions (arm region, trunk region, leg region, shoulder and neck region). Pressure pain threshold were calculated by summarizing PPT [kg] of specific muscles for each body region. To evaluate muscle strength, MRC or dynamometer values were used. For the arm and leg region, muscle strength was evaluated by summarizing dynamometer values [kg] for legs respectively arm muscles. For the neck and trunk region, MRC (Medical Research Council) scale was specified for the neck extensor muscles and the muscles of the trunk by assigning a value from 0 (complete paralysis) to 5 (normal strength)

*Significance at a level ≤ 0.05

All significant values are bold

#### Parameters of the MyotonPro

Correlation with the parameters of the MytonPro^®^ are presented in Table 6. In a Kendall’s Tau correlation for the PPT of each muscle and their corresponding MyotonPro^®^ parameter, such as frequency, stiffness, decrement, relaxation and creep, only the PPT of the rectus femoris on the right side correlated negatively with its decrement (*p* = 0.044, *r* = − 0.349). The regression model was not significant (*R* = 0.368, *R*^2^ = 0.135, *p* = 0.133).

**Table 6:** Significance of correlations (Kendall’s Tau) between PPT values with their corresponding myoton parameters (frequency, stiffness, decrement, relaxation, creep) in the same muscle

Corresponding PPT and MyotonPro parameters	Frequency	Stiffness	Decrement	Relaxation	Creep
Neck extensor right Neck extensor left	0.268 0.891	0.140 0.585	0.853 1.000	0.538 0.750	0.712 0.339
Deltoid right Deltoid left	0.342 0.618	0.210 0.648	0.255 0.676	0.224 0.835	0.270 0.803
Biceps brachii right Biceps brachii left	0.467 0.879	0.423 0.425	0.360 0.380	0.593 0.424	0.674 0.254
Supraspinatus right Supraspinatus left	0.433 0.402	0.563 0.483	0.303 0.117	0.563 0.364	0.301 0.386
Trapezius right Trapezius Left	0.964 0.116	0.752 0.231	0.964 0.215	1.000 0.200	0.822 0.200
Erector spinae right Erector spinae left	0.221 0.786	0.557 1.000	0.442 0.443	0.684 0.717	0.964 0.588
Rectus femoris right Rectus femoris left	0.240 0.053	0.596 0.240	0.044* 0.211	0.289 0.053	0.362 0.069
Tibialis anterior left Tibialis anterior right	0.359 0.650	0.342 0.155	0.386 0.836	0.342 0.302	0.277 0.252
Gastrocnemius right Gastrocnemius left	0.569 0.836	0.791 0.741	0.068 0.117	0.733 0.901	0.543 0.967

All significant values are bold

#### 6MWT

Results of the adapted 6MWT in 13 patients are shown in Table 7. There was no significant difference in the distance walked between women and men. Only two women experienced pain during the 6MWT. A comparison of PPT values before and after the 6MWT of the rectus femoris and the gastrocnemius muscles showed a slight increase in men, reflecting less pressure pain. In contrast, a slight decrease was found in women. The pre–post difference was only statistically significant for the rise in the right rectus femoris muscle for men (*p* = 0.034), and further analysis using Cohen’s *d* suggests a high effect size (*d* = 0.79).

**Table 7:** 6MWT and PPT values before and after physical exertion

	Men (*N* = 13)	Women (*N* = 7)	Women and men (*N* = 20)
6MWT performed	61.5% (*N* = 8)	71.4% (*N* = 5)	65% (*N* = 13)
6MWT meters			
Mean	397.5	443.6	415.23
Median	452.5	466	466
Sensation of pain during 6MWT	0% (*N* = 0)	28.6% (*N* = 2)	(*N* = 2)
Rectus femoris muscle:			
PPT before 6MWT right/left [kg]	6.29/6.14	4.22/3.84	5.49/5.25
PPT after 6MWT right/left [kg]	7.03/6.64	3.48/3.52	5.66/5.44
Difference right/left [kg]**	+ 0.74/ + 0.5	− 0.74/− 0.32	+ 0.17/ + 0.19
Difference right/left [%]	+ 11.8%/ + 8.1%	− 17.5%/− 8.3%	+ 3.1%/ + 3.6%
p* right/left	0.034*/0.265	0.051/0.246	0.562/0.530
Cohens *D*	0.79/-	−/−	−/−
Gastrocnemius muscle:			
PPT before 6MWT right/left [kg]	5.43/5.35	3.82/3.36	4.82/4.58
PPT after 6MWT right/left [kg]	5.45/5.35	3.80/3.22	4.82/4.53
Difference right/left [kg]**	+ 0.02/ + 0.00	− 0.02/− 0.14	+ 0.00/− 0.05
Difference right/left [%]	+ 0.00%/ + 0.00%	− 0.00%/− 4.2%	± 0.0%/− 1.09%
p* right/left	0.97/1.00	0.936/0.611	1.00/0.812
Cohens *D*	−/−	−/−	−/−

*Results of PPT were not normally distributed (Shapiro–wilk test); group differences were assessed by a paired *t* test. Bold values indicate significant difference for *p* ≤ 0.05. Gastrocnemius PPT test results were not normally distributed, group differences were assessed by Wilcoxon signed rank test. **Positive higher values reflect less pressure pain

### Study limitations

Despite the relatively high number of SMA patients regularly seen at the neuromuscular expert centre, only a small number of patients were eligible or consented to participation in this study, making a comprehensive statistical analysis of factors influencing nociceptive pain not reasonable. On the other hand, this is the first study in a sufficient number of patients with longstanding SMA3 evaluating nociceptive pain by combining results from clinical assessments, medical history and pain-related questionnaires.

## Discussion

We conducted this pilot study of nociceptive pain in longstanding SMA 3 (walkers) patients, which is the first to our knowledge that combines results from medical history, clinical assessments and pain-specific and health-related patient-reported outcomes. In summary, muscle pain in SMA3 is reported by approximately 50% of the patients and musculoskeletal dysbalance due to regional muscle weakness seems to be the underlying cause. Due to our findings, muscle pain is associated with muscular weakness in the arms and legs, but not with changes in muscular stiffness, frequency, decrement, relaxation or creep.

Measurements of pressure pain threshold (PPT) were lower in women than in men in nearly all muscles, indicating a higher susceptibility of pain perception in women. In healthy volunteers, examinations [7] showed that male sex and higher age are associated with higher PPTs in some muscles. However, differences between men and women were only statistically significant in the rectus femoris muscle on the left side (*p* = 0.037; supplements). Besides, we found an association between muscle strength and algometer scores of the arms and legs in the linear regression model (Table 2), especially in distal limbs. A lower score in dynamometry was associated with lower scores in PPT, which reflects a higher muscular sensitivity to pressure in weaker muscles.

Assessments of pressure pain threshold (PPT) before and after the 6-min walk test, which reflects a standard test for muscular endurance in neuromuscular disorders, did not show valuable or clinical meaningful change. Of the cohort, only two women complained of muscular pain after the 6MWT. Besides, patients reported a way of pain relief due to muscle exercise, but also a high number complained about muscle pain after too much exercise and physical exertion. Only two female patients used pain-specific medication. Although conclusions should be made very carefully due to the small sample size, our results do not indicate that muscle endurance activity measured by the 6MWT induces muscle pain or lowers the pain threshold. As reported by patients, muscular endurance exercises may be more beneficial for pain reduction than provoking pain, as long as muscles are not overstressed. Overall, further research is necessary for the efficacy of muscle training in SMA patients to prevent muscle wasting and muscle pain and maintain muscle strength as long as possible.

We did not find correlations between the PPT values and the MyotonPro values for frequency, stiffness, decrement, relaxation and creep in the corresponding muscles. The only statistically significant correlation was found between the PPT of the rectus femoris on the right side and the decrement of the right side (Kendall’s Tau *p* = 0.044, correlation coefficient = − 0.349; *R*^2^ = 0.135). PPTs of the rectus femoris muscle decrease with higher decrement values equivalent to lower elasticity values (decrement is inversely proportional to elasticity). Elasticity is a measure of the muscle’s ability to recover its initial shape after deformation. The lower elasticity of the muscle, the lower the PPTs.

Although women experienced pain significantly more often and with a higher severity, they experienced a higher habitual well-being and lower scores for depression, anxiety and stress than men experiencing pain. Thus, the higher perception of pain in women does not appear to be affected by these psychological comorbidities. Musculoskeletal comorbidities occurred in both men and women (scoliosis and cervical spine stenosis), but could not explain the high proportion of low back pain in women.

Comparison with results of previous studies is difficult to make, as most of them were questionnaire based and none included objective clinical assessments. A survey from de Groot et al. [5] summarized physical complaints in longstanding SMA type 3 patients and a severe disease course to determine whether new symptoms arise with increased age. The high prevalence of fatigue of 61.4% corresponds to our findings, but the prevalence of muscle pain was significantly higher in our cohort (31.4% vs 55%). Considering the results of both studies, back pain (27.1%) and neck pain (34.3%) were essential complaints of SMA3 patients (Figs. 1and 2). In contrast, a survey from Abresch et al. [2] evaluated pain profiles of various neuromuscular diseases, including 29 patients with SMA3. As the main result of this survey, the prevalence of pain and pain sensitivity evaluated for the SMA type 2 and type 3 group was not significantly different from a US population (30.2% in the SMA group vs 31.8% in the US population reporting good health and no pain). In the SMA group, the pain was affected by gender, which fits the results of our study reporting a higher pain prevalence in female patients.

Even though a potential effect on pain of nusinersen was not a predefined outcome, our data suggest that there is no relevant impact on the occurrence or frequency or intensity of pain, which might be due to a relatively short-term specific therapy in longstanding chronic disease. But further particular studies are needed to evaluate the effect of SMA specific therapies on nociceptive pain.

Overall, muscle pain in SMA type 3 cannot be explained by changes in muscular stiffness, frequency, decrement, relaxation or creep. This shows that muscle pain in SMA type 3 is mainly caused by changes in the dysbalanced musculoskeletal system, caused by (regional) muscular weakness. As our conclusions are based on a relatively small cohort, we suggest evaluating pain in SMA3 in larger cohorts in the future.

## Supplementary Information

Below is the link to the electronic supplementary material.

Supplementary file1 (DOCX 18 KB)

## Data Availability

The anonymized participant data presented here are available upon request from the correspondent author (stephan.wenninger@med.uni-muenchen.de).
