# Supplementary material for: Nociceptive pain in adult patients with 5q-spinal muscular atrophy type 3: a cross-sectional clinical study
Source: J Neurol. 2022 Aug 29;270(1):250–61. doi: 10.1007/s00415-022-11351-0 (PMC9813071; doi:10.1007/s00415-022-11351-0)
Supplement: Supplementary file 1 — Supplementary file1 (DOCX 18 KB) [file 415_2022_11351_MOESM1_ESM.docx]

**Supplements**

**Table 1** Mann Whitney U Test of the PPT scores divided by gender; all mean(±SD; min; max);

|  | Men(n=13) | Women(n=7) | p |
| --- | --- | --- | --- |
| PPT M. biceps brachii left  PPT M. biceps brachii right | 3.05(±0.89; 1.8; 4.8)  2.92(±0.92; 1.7; 4.8) | 2.64(±0.80; 1.9; 4.3)  2.84(±0.76; 2.0; 4.0) | 0.311  1.000 |
| PPT M. deltoideus left  PPT M. deltoideus right | 3.99(±1.38; 2.5; 6.8)  4.15(±1.51; 2.1; 7.3) | 2.96(±0.77; 1.8; 4.1)  2.91(±0.63; 2.0; 3.7) | 0.115  0.056 |
| PPT M. trapezius left  PPT M. trapezius right | 4.36(±2.22; 2.1; 8.3)  4.13(±1.74; 2.3; 7.5) | 3.24(±0.80; 2.1; 4.3)  3.20(±0.87; 2.0; 4.5) | 0.588  0.438 |
| PPT M. supraspinatus left  PPT M. supraspinatus right | 4.19(±1.50; 2.2; 7.5)  4.61(±1.92; 2.5; 9.0) | 3.49(±0.96; 2.1; 4.8)  3.66(±1.06; 2.4; 5.1) | 0.432  0.299 |
| PPT Neck extensor | 3.23(±1.25; 1.9; 6.4) | 2.20(±1.05; 0.4; 3.4) | 0.149 |
| PPT M. erector spinae left  PPT M. erector spinae right | 5.59(±2.02; 3.3; 10)  6.01(±1.98; 3.8; 10) | 4.47(±1.68; 2.3; 6.8)  4.55(±1.59; 2.4; 6.0) | 0.256  0.149 |
| PPT M. rectus femoris left  PPT M. rectus femoris right | 5.12(±1.61; 2.2; 8.3)  5.59(±1.91; 2.7; 9.0) | 3.71(±0.95; 2.4; 5.0)  4.29(±1.65; 2.5; 7.0) | 0.037*  0.135 |
| PPT M. tibialis anterior left  PPT M. tibialis anterior right | 4.78(±2.00; 2.2; 8.8)  5.23(±1.86; 2.8; 9.4) | 4.85(±2.04; 3.0; 8.7)  4.49(±0.82; 3.1; 5.5) | 1.000  0.536 |
| PPT M. gastrocnemius left  PPT M. gastrocnemius right | 5.03(±2.37; 2.2; 9.0)  5.17(±2.46; 1.9; 9.5) | 3.95(±1.39; 2.2; 6.3)  4.69(±1.44; 2.5; 6.6) | 0.494  1.000 |
